# Relevance of physicochemical properties and functional pharmacology data to predict the clinical safety profile of direct oral anticoagulants

**DOI:** 10.1002/prp2.603

**Published:** 2020-06-04

**Authors:** Charles J. Ferro, Fay Solkhon, Zahraa Jalal, Abdullah M. Al‐Hamid, Alan M. Jones

**Affiliations:** ^1^ Queen Elizabeth Hospital University Hospitals Birmingham NHS Foundation Trust Birmingham UK; ^2^ School of Pharmacy University of Birmingham Birmingham UK

**Keywords:** DOACs, risk factors, selectivity profile, Yellow Card

## Abstract

Direct oral anticoagulants (DOACs) have rapidly become the drug class of choice for anticoagulation therapy in secondary care. It is known that gastrointestinal hemorrhage are potential side effects of the DOAC drug class. In this study we have investigated the relevance of molecular structure and on/off‐target pharmacology as a predictor of adverse drug reactions (ADRs) for the DOAC drug class. Use of the Reaxys MedChem module allowed for data mining of all possible reported off‐target effects of the DOAC class members. For the first time, the MHRA Yellow card database in combination with prescribing rates in the United Kingdom (data for n = 30 566 936 DOAC *R_x_* (up to 2017) and ADR data n = 22 275 (up to 2018)) were used for our data comparison of DOACs. From the underlying reported data, we were able to rank the DOACs in terms of the likely adverse events we would expect to observe. We identified potential risks of ADRs based on the DOACs pharmacology including the expected GI hemorrhage, but also the unexpected risk of stroke, pulmonary embolism and kidney injury. Statistically significant (*P* < .001) differences were found between all DOACs and their total number of ADRs. Although the risks are small, strong statistical correlation between observed pharmacology and national ADR data is observed in three out of the five areas of concern.

**Abbreviations**
ADRs: adverse drug reactions
DOACs: Direct oral anticoagulants
PMR: postmarketing surveillance

## 1 INTRODUCTION

Adverse drug reactions (ADRs), defined as all noxious and unintended responses to a medicinal product related to any dose, are common affecting 10%‐20% of hospitalized patients and up to 25% of outpatients.^1^ As such they are a major burden on healthcare systems and there is a pressing need to understand both patient and drug‐related risk factors and mechanisms that contribute to these events. Limited access to large and diverse patient populations in clinical trials, untested drug co‐administrations, and longer term treatments often result in post‐marketing labeling and occasional withdrawals.^2^ Furthermore, the occurrence of off‐target ADRs are not predictable based on the drug's specific therapeutic effect.^1^ Therefore, postmarketing surveillance (PMR) schemes have been introduced in many countries and internationally to improve patient safety by identifying previously unrecognized ADRs, drug interactions, and patient subgroup vulnerability.^3^, ^4^, ^5^ However, these schemes are reactive rather than proactive.

Direct oral anticoagulants (DOACs) include the sole direct thrombin inhibitor (Dabigatran) and three direct factor Xa inhibitors (Rivaroxaban, Apixaban and Edoxaban) were first licensed in the United Kingdom in 2008 (Dabigatran), 2012 (Rivaroxaban and Apixaban), and 2015 (Edoxaban), respectively. DOACs are currently used for the treatment of venous thrombo‐embolism (VTE), thromboprophylaxis, prevention of stroke, and embolization in nonvalvular atrial fibrillation (AF) and are rapidly becoming the drug class of choice for anticoagulation therapy, superseding warfarin.^6^, ^7^ DOACs are preferred for their ease of use, favorable pharmacokinetics, fewer reported drug interactions, and lack of monitoring requirements.^8^ Clinical trials have identified that DOACs have very similar efficacy profiles compared to warfarin and are noninferior, resulting in lower incidences of some major bleeds. In contrast, DOACs are associated with higher risks of gastrointestinal hemorrhage (GI) when compared to warfarin.^9^, ^10^, ^11^, ^12^ There are concerns regarding other potentially serious ADRs linked with the DOAC drug class and significant mortality rates, such as liver impairment and central nervous system disorders.^7^, ^13^, ^14^, ^15^, ^16^, ^17^, ^18^

Recent studies have revealed that drugs believed to be highly selective frequently address multiple target proteins.^19^ In this study we considered whether the on‐ and off‐target selectivity profiles^1^, ^2^, ^20^, ^21^, ^22^, ^23^, ^24^, ^25^, ^26^ for a drug class with a novel mechanism of action (DOACs) could inform or predict the likelihood of serious adverse events within a real‐world population. This research aims to identify ADRs of the four DOACs currently available in the United Kingdom, and to discover whether any of these could have potentially been predicted by linking back to their molecular properties.

### 1.1 Aims and objectives

To predict potential adverse drug reactions based on the identified DOAC chemical properties and pharmacology. To identify if the predicted adverse drug reactions are statistically relevant.

## 2 MATERIALS AND METHODS

### 2.1 Chemical properties and pharmacology

Reaxys^®^ Medicinal Chemistry database was used to characterize the chemical properties, including metabolism/clearance pathways, volume of distribution (Vdss) and plasma protein binding (PPB), and selectivity profiles for all four DOACs.^27^ How drugs are cleared from the body can give an indication of areas for ADRs to occur.^28^ Full results are included in Table S1. In particular the following parameters of interest were extracted:

#### 2.1.1 Target affinity

To facilitate comparisons of data from different publications, Reaxys^®^ medicinal chemistry uses *p*X values. These are normalized values assigned to the data to enable quantification of the substance‐target affinity and comparison of disparate information. This allowed for a systematic interrogation of the available literature, combined with identifying the primary literature source to convert the *p*X value into a relatable *p*IC_50_ (Negative log of the half maximal inhibitory concentration (IC_50_ value), when converted to molar) value. A threshold of ~10 μmol/L (*p*X = 5) for at least one DOAC was used to exclude biologically insignificant off‐target interactions.^19^ Extracted *p*X data were compared to the primary literature using SciFinder, PubMed, Reaxys, EMBASE, and PubChem databases.

#### 2.1.2 Molecular obesity and on‐target efficiency metrics

Molecular obesity is the term for the anticipated degree of nonspecific interacting lipophilic components of a drug based on the correlation of logP (where *P* is the partition coefficient = concentration of solute in octanol divided by the concentration of solute in water)^29^, ^30^ with promiscuity of thousands of pharmaceutical entities. High molecular weight and logP are correlated to the propensity of a drug having more off‐target behavior.^30^, ^31^

The Lipophilic Ligand Efficiency (LLE) index, a measure of how efficient a drug binds to its target excluding nonspecific entropic factors was calculated using LLE = *p*IC_50_ − log P. The parameter LLE is considered an important parameter to normalize potency relative to lipophilicity.^32^, ^33^ A value of less than five is suggested to be associated with an increased risk of toxicity for any given compound.^31^

#### 2.1.3 Blood‐brain barrier penetration

The risk of neurological ADRs is markedly increased if a drug can penetrate the blood brain barrier (BBB). Thresholds for BBB penetration are as follows: molecular weight < 450 Da; <6 hydrogen bond donors (HBD); <2 hydrogen bond acceptors (HBA); Neutral or basic drug molecule as defined by the *p*K_a_ (negative base10 logarithm of the acid dissociation constant; the lower the *p*K_a_ value the stronger the acid); topological polar surface area (tPSA) <90 Å; logD_7.5_ 1‐3 (logD is the distribution constant of a drug between the aqueous and lipid phases at *p*H 7.5); and low affinity to efflux P‐glycoprotein mechanisms. The more of these properties possessed by a drug the greater the likelihood of BBB penetration.^34^, ^35^

### 2.2 Yellow Card Scheme

The Yellow Card Scheme run by the Medicines and Healthcare Products Regulatory Agency (MHRA), administers the collection of data relating to suspected ADRs in the United Kingdom. It was devised to improve patient safety and identification of adverse patient events in 1964 in the wake of the thalidomide disaster.^5^ Adverse patient event data were collected from the Yellow Card Scheme Interactive Drug Analysis Profiles web portal (https://yellowcard.mhra.gov.uk/iDAP/) up to November 2018.^36^ The spontaneous reporting system had processed information from 2008 up to October 2018.

### 2.3 Prescribing data

UK prescribing data were collected from Open Prescribing database (https://openprescribing.net/) and provide data on all National Health Service (NHS) prescribing in English primary care.^37^, ^38^

### 2.4 Prediction approach used

Meeting the chemical properties (eg, LLE < 5 for general ADR risk) and pharmacology definitions (eg, *p*X > 5 for specific ADR risks) outlined above were used as predictors of ADRs. The use of off‐target inhibition data to predict ADRs was used with the caveat of biologically relevant levels of inhibition (<10 µmol/L) for the target of interest. If a drug had potent biological inhibition of an off‐target protein, the protein's biological relevance was investigated via a separate literature search algorithm. Bringing together these disparate information sources generated possible ADR effects.

### 2.5 Statistical analysis

Data were tabulated from the above sources. Chi‐squared tests were performed in order to determine statistical significance between ADRs associated with each DOAC, using IBM SPSS 24. A *P* value of less than .05 was considered statistically significant. Because of the exploratory nature of this study, the lack of data on potential confounders, and the relatively low incidence of some of the ADRs, we used raw data rather than disproportionate analysis. The exploratory nature of this study also meant that corrections for multiple comparisons were not used.

### 2.6 Ethics approval and consent to participate

This study uses publicly available data and no individual level data. It therefore does not require ethical approval.

## 3 RESULTS

### 3.1 Chemistry and pharmacology data

#### 3.1.1 Chemical properties and pharmacology

The chemical properties and pharmacology of the DOACs are summarized in Table 1. Although all four drugs are termed DOACs, Dabigatran has a unique mode of action targeting Thrombin (IC_50_: 1.2 nmol/L); Apixaban, Rivaroxaban, and Edoxaban all target Factor Xa (IC_50_ 0.08, 0.4 and 0.56 nmol/L respectively), in the clotting cascade. All DOACs show selectivity for their molecular target (Factor Xa or thrombin) with modest off‐target effects for thrombin/Factor Xa and vice versa. Dabigatran is the only DOAC formulated as a prodrug form (Dabigatran etexilate).

**TABLE 1:** Physicochemical, blood‐brain barrier, pharmacokinetic and pharmacological properties of the four evaluated drugs

Variable	Apixaban	Dabigatran^a^	Edoxaban	Rivaroxaban
Molecular obesity and on‐target efficiency metrics				
log *P*	2.02	2.05	0.89	1.35
*p*IC_50_ (main target)	10.1	8.9	9.3	9.4
LLE	8.1	6.9	8.4	8.0
Blood‐brain barrier penetrant properties				
MW (Da)	459.5	471.5	548.1	435.9
*p*K_a_	Neutral	4.25	7.37	neutral
tPSA, (Å)	108.54	147.47	135.57	88.18
HB acceptors	5	7	8	6
HB donors	1	4	3	1
clog D_7.5_	1.89	−1.21	0.28	2.39
P‐glycoprotein substrate	Yes	Yes	Yes	Yes
No. of BBB requirements met	**4**	**1**	**2**	**5**
Pharmacokinetics				
Bioavailability	50%	6.5%	62%	80%
Half‐life (hours)	12	8.8	9‐11	6‐8
Liver CYP450 metabolism	Yes	Yes	Yes	Yes
CYP_3_A_4_ substrate	Yes (~25%)	No	No	Yes (33%)
Renal excretion	25%	80%	35%	67%
Volume of distribution	21 L	60‐70 L	107 L	50 L
PPB	87%	35%	55%	95%
Dosing	BID	OD‐BID	OD	OD‐BID
On‐ and off‐target activities				
Factor Xa (nmol/L)	0.08	3760	0.561	0.4
Thrombin (nmol/L)	3100	1.2	6000	1000
Factor VIIa (nmol/L)	>15 000	—	41 700	>20 000
Factor IXa (nmol/L)	>15 000	—	—	—
Factor XIa (nmol/L)	—	—	—	>20 000
Plasmin (nmol/L)	>25 000	1695	—	>20 000
Plasma Kallikrein (nmol/L)	3700	—	—	—
APC (nmol/L)	>30 000	—	—	>20 000
tPA (nmol/L)	<40 000	45 360	—	—
Trypsin (nmol/L)	4200	50.3	—	>20 000
Chymotrypsin (nmol/L)	3500	—	—	—
NQ02 (nmol/L)	—	10 000	—	—
Matriptase (nmol/L)	—	—	—	3350
Hepsin (nmol/L)	—	835	—	—

Abbreviations: APC, allophycocyanin; clogD_7.5_, calculated logD at *p*H = 7.5 (where D is the distribution coefficient = concentration of solute in octanol divided by the concentration of solute in water); HB, hydrogen bond; LLE, lipophilic ligand efficiency; NQ02, Quinone oxidoreductase; tPA, tissue plasminogen activator.

^a^ Figures given for active drug only.

#### 3.1.2 Molecular Obesity and risk of off‐target effects

Properties of the DOACs that relate to on‐target efficiency (logP, IC_50,_ and LLE) are shown in Table 1. Dabigatran has the highest logP (5.26) as its prodrug etexilate form and as the active drug (2.05). Dabigratran has the lowest LLE as both the active drug (6.9) and prodrug (3.7) with the other three DOACs having very similar LLE values (8.0‐8.4). From this holistic measure it can be inferred that Dabigatran should have the least clean off‐target profile.

#### 3.1.3 Blood‐brain barrier penetrant properties

The seven properties of the DOACs relevant to the risk of BBB penetration are shown in Table 1. Taken in turn, Rivaroxaban meets the MW requirement (435.9 Da) and Apixaban and Dabigatran are on the borderline (459 and 471 Da, respectively). Apixaban and Rivaroxaban met the requirement for less than six HBAs and less than two HBDs. All DOACs are neutral or basic in the given form, with only the active form of Dabigatran being an acid. Only Rivaroxaban meets the tPSA requirement (88 Å). Apixaban and Rivaroxaban meet the logD_7.5_ requirements at 1.89 and 2.39 respectively. All DOACs are P‐gp substrates. Based on these results we would estimate that the greatest risk for BBB penetration would be with Rivaroxaban (5 out of 7) followed by Apixaban (4 out of 7). Edoxaban (2 out of 7) and Dabigatran (1 out of 7) would have the lowest potential risk for BBB penetration.

#### 3.1.4 Pharmacokinetic information and relationship to potential ADRs

The pharmacokinetic profile of the DOACs are shown in Table 1. Dabigatran (80%) has the highest renal clearance followed by Rivaroxaban (67%‐33%). Importantly, Dabigatran is the only acidic DOAC (*p*K_a_ = 4.25), Dabigatran etexilate is also formulated as a tartaric acid salt increasing the localized acidic nature of this DOAC which may have implication in resulting GI ADRs compared to Apixaban, Edoxaban, or Rivaroxaban.

#### 3.1.5 Key off‐target biological receptors identified in the study and links to disease stages

As expected, close members of the clotting cascade inhibition data were widely reported (Table 1).

Apixaban has a high selectivity for Factor Xa over thrombin (>38750‐fold) and other Factor X isoforms. Potentially clinically significant off‐targets at a much lower efficacy than the on‐target (Factor Xa) are plasma Kallikrein (3700 nmol/L), trypsin (4200 nmol/L), and chymotrypsin (3500 nmol/L), all protein degradation enzymes in the GIT. The reported tPA data for Apixaban is not a specific value: <40 μmol/L. Inhibition of tPA has been linked to pulmonary embolism.^39^, ^40^

Dabigatran has good selectivity for thrombin over Factor Xa (>3133 fold) but a significant number of potentially biologically relevant off‐targets: plasmin (1700 nmol/L), tPA (45 μmol/L), chymotrypsin (10 000 nmol/L), NQ02 (10 000 nmol/L), hepsin (835 nmol/L), and most potently, trypsin (50 nmol/L) which is 42‐fold from the desired on‐target.

Edoxaban has a good selectivity profile for Factor Xa (0.5 nmol/L) over thrombin (>10 600‐fold) and Factor X isoforms (>74 330‐fold). At the time of writing, no nonclotting cascade off‐target biological data have been disclosed in the literature. Edoxaban therefore, has the cleanest off‐target profile from the available dataset.

Rivaroxaban has a good selectivity profile for Factor Xa over thrombin (2500‐fold) and the only potentially significant off‐target affinity being matriptrase (3400 nmol/L). Matriptrase being similar in function to trypsin in the GI tract.^41^

Based on the on‐/off‐target profiles it would be expected that Dabigatran, followed by Apixaban, Rivaroxaban, and finally Edoxaban have the greatest number of ADRs overall. The off‐target potency data for Dabigatran, Apixaban, and Rivaroxaban all point toward an increased risk of GI ADRs. Taken together with Dabigatran's acidic formulation, it would be expected that Dabigatran would have the highest propensity of all the DOACs for a GI complication. Apixaban could potentially be associated with a higher risk of pulmonary embolism.

### 3.2 Overall summary of predictions based on chemistry and pharmacology data

1. Based on the molecular obesity and on‐/off‐target profiles it would be expected that, Dabigatran, would have the greatest number of ADRs overall.
2. Based on renal clearance it would be expected that, in decreasing order, Dabigatran, Rivaroxaban, Edoxaban, and Apixaban would have the greatest number of renal ADRs.
3. Based on off‐target inhibition of gut proteases and formulations it would be expected that Dabigatran and Apixaban would have the greater number of GI ADRs.
4. Based on the BBB penetrant molecular properties it would be expected that Rivaroxaban and Apixaban would be associated with the greater number of CNS ADRs.
5. Based on off‐target tPA inhibition, it would be expected that Dabigatran and Apixaban would be associated with a greater number of PE ADRs.

### 3.3 Prescription and adverse drug reactions data

Data obtained from the Yellow Card reporting scheme and NHS prescribing data were analyzed to confirm or refute the predictions formulated above based on the chemical and pharmacological properties of the individual DOACs.

The number of prescriptions for each DOAC in the United Kingdom (up to 2017 for lag detection of ADRs) and number of reported ADRs (2008‐2018) and fatalities within the United Kingdom are shown in Table 2 together with the *P*‐value for comparison across all the DOACs. For the purpose of this research, it was estimated that an average of 1 year from when the ADR occurred in a patient and when it appears on the Yellow Card Scheme drug analysis profiles was an appropriate lag‐time which is also the finest level of granularity possible with the data available. The individual *P*‐values for differences between the individual DOACs are shown in Table S2. A total of 30 566 936 DOAC prescriptions and 22 725 ADR reports were analyzed. Rivaroxaban had the most prescriptions issued in the timeframe of this study, followed by Apixaban, Dabigatran, and Edoxaban.

**TABLE 2:** Summary of the reported adverse drug reactions associated with all four currently available DOACs in the UK. Numbers in brackets are per 100 000 prescriptions. *P*‐values obtained by Chi‐square analysis detailed in the supporting information

	Apixaban	Dabigatran	Rivaroxaban	Edoxaban	*P*‐value
Total prescriptions	8 471 045	2 059 119	10 741 318	295 454	
Total ADRs	5874 (69)	4050 (197)	11 860 (110)	385 (130)	<0.001
Fatalities	200 (2.36)	163 (7.92)	368 (3.42)	10 (3.38)	<0.001
Renal system					
Total ADRs	169 (2.00)	139 (6.75)	389 (3.62)	8 (2.71)	<0.001
Fatalities	3 (0.04)	0 (0)	2 (0.02)	0 (0)	0.756
AKI	29 (0.34)	33 (1.60)	48 (0.45)	0 (0)	<0.001
Gastrointestinal system					
Total ADRs	1051 (12.41)	1138 (55.27)	2309 (21.50)	81 (27.42)	<0.001
Fatalities	29 (0.34)	38 (1.85)	52 (0.48)	1 (0.34)	<0.001
GI Hemorrhage	461 (5.44)	427 (20.74)	1033 (9.62)	27 (9.14)	<0.001
Fatal GI Hemorrhage	30 (0.35)	38 (1.85)	51 (0.47)	1 (0.34)	<0.001
Central nervous system					
Total ADRs	977 (11.53)	482 (23.41)	1709 (15.91)	64 (21.66)	<0.001
Fatalities	92 (1.09)	37 (1.80)	177 (1.65)	3 (1.02)	0.005
Stroke	97 (1.15)	64 (3.11)	102 (0.95)	10 (3.38)	<0.001
Fatal stroke	15 (0.18)	5 (0.24)	17 (0.16)	0 (0)	0.742
Hemorrhagic stroke	25 (0.30)	5 (0.24)	42 (0.39)	0 (0)	0.403
Respiratory system					
Total ADRS	378 (4.46)	259 (12.58)	836 (7.78)	14 (4.74)	<0.001
Fatalities	5 (0.06)	10 (0.49)	17 (0.16)	0 (0)	<0.001
Pulmonary embolism	57 (0.67)	75 (3.64)	131 (1.22)	2 (0.68)	<0.001

#### 3.3.1 Overall adverse drug reactions and fatalities

Overall, Dabigatran (0.20%) had the highest percentage of ADRs relative to the total number of prescriptions followed by Edoxaban (0.13%), Rivaroxaban (0.11%), and Apixaban (0.07%). Standardizing for a typical daily dose equivalent (DDE) the following trend emerged in the percentage of ADRs reported; Edoxaban (0.13%), Rivaroxaban (0.06%‐0.11%), Dabigatran (0.10%), and Apixaban (0.02%‐0.04%). The high percentage ADRs for Edoxaban are likely due to its black triangle status. A similar trend was observed for fatalities with Dabigatran having the most (7.92%) and Apixaban (2.36%) the least proportionate to the number of prescriptions (Edoxaban (3.42%), Rivaroxaban (3.38%)). On standardizing for DDE fatalities the following trend is observed; Dabigatran (3.46%), Edoxaban (3.42%), Rivaroxaban (1.69%‐3.38%), and Apixaban (0.59%‐1.18%). Due to complications resulting from how the DOACs are prescribed depending on the indication ranging from 1, 2, or 4 tablets per day, the following data are normalized to the number of prescription items.

#### 3.3.2 Renal adverse drug reactions and fatalities

The highest risk of renal ADRs was associated with Dabigatran, Rivaroxaban, Edoxaban, and Apixaban in descending order. The major reported renal ADR was acute kidney injury (AKI) accounting for 23.7% of all renal ADRs. The highest risk of AKI occurred with Dabigatran (1.60 per 100 000 prescriptions), Rivaroxaban (0.45), and Apixaban (0.34) with no AKI reports associated with the use of Edoxaban. There were five renal fatalities attributed to DOACs during the timeline of this retrospective study.

#### 3.3.3 Gastrointestinal adverse drug reactions and fatalities

For all DOACs, the gastrointestinal tract (GIT) was the organ with the highest reported ADRs. Dabigatran had a GI ADR rate of 55.27 per 100 000 prescriptions, significantly higher than Edoxaban (27.42), Rivaroxaban (21.50), or Apixaban (12.41).

Within GI hemorrhage, Dabigatran recorded the highest number of ADRs at 20.74 per 100 000 prescriptions, followed by Rivaroxaban (9.62), Edoxaban (9.14), and Apixaban (5.44). These ADR levels bear out within the GI hemorrhage fatality figures at 1.85 per 100 000 prescriptions for Dabigatran, followed by Rivaroxaban (0.47), Apixaban (0.35), and Edoxaban at 0.34 (on the basis of a single fatality).

#### 3.3.4 Central nervous system adverse drug reactions and fatalities

For all DOACS, the CNS was the organ system with the second highest reported ADRs. Dabigatran had the highest proportion of reported CNS ADRs with 23.41 per 100 000 prescriptions, followed by Edoxaban (21.66), Rivaroxaban (15.91), and Apixaban (11.53). Dabigatran, Rivaroxaban, and Apixaban had the highest number of fatal outcomes within the central nervous system category at 1.80, 1.09, respectively per 100 000 prescriptions (Edoxaban, 1.02).

Edoxaban had the highest number of stroke ADRs per 100 000 prescriptions at 3.38, followed by Dabigatran (3.11), Apixaban (1.15), and Rivaroxaban (0.65). A different trend emerged within the fatal stroke category with Dabigatran being the highest at 0.24 per 100 000 prescriptions, followed by Apixaban (0.18) and Rivaroxaban (0.16). No stroke fatalities were attributed to Edoxaban.

Rivaroxaban had the highest proportion of hemorrhagic strokes at 0.39 per 100 000 prescriptions, followed by Apixaban (0.30) and Dabigatran (0.24). None were reported for Edoxaban.

#### 3.3.5 Respiratory system adverse drug reactions and fatalities

Dabigatran had the highest number of reported ADRs associated with the pulmonary system at 12.58 per 100 000 prescriptions followed by Rivaroxaban (7.78), Edoxaban (4.74), and Apixaban (4.46).

Overall there were 32 respiratory system deaths attributed to DOACs with Dabigatran having the highest proportion at 0.49 per 100 000 prescriptions followed by Rivaroxaban (0.16) and Apixaban (0.06) with no respiratory deaths attributed to Edoxaban.

For pulmonary embolism, Dabigatran had the highest number per 100 000 prescriptions at 3.64, followed by Rivaroxaban (1.22), and Edoxaban (0.68), and Apixaban (0.67).

## 4 DISCUSSION

Based on the chemistry and pharmacology data we obtained from all four DOACs, we made several predictions on the ADRs that would have been reported for each individual DOAC. In general terms, our predictions held true supporting our initial premise that the chemical and pharmacological properties of each DOAC could be used to guide targeted postmarketing drug surveillance.

### 4.1 Overall adverse drug reactions and fatalities

Based on the chemical properties relating to on‐target efficiency (logP, IC_50,_ and LLE) we predicted that Dabigatran would have the least clean off‐target profile. This was indeed the case with Dabigatran having several off‐target activities in the micromolar range. Our prediction that Dabigatran would have the most overall ADRs also held true with a rate of 197 ADRs per 100 000 prescriptions compared to the next highest, Rivaroxaban, with a rate of 110 per 100 000 prescriptions. This finding was also supported by Dabigatran having the highest rate of fatalities reported at 7.92 per 100 000 prescriptions. This is more than double the next highest, Rivaroxaban at 3.42 per 100 000 prescriptions crudely suggesting that the severity, and not just the frequency, of ADRs is highest with Dabigatran out of the four DOACs. Dabigatran is currently the least prescribed of the three established DOACs, Edoxaban has been licensed only since 2015 and is under Black Triangle reporting status with the MHRA. This lower prescribing rate has been attributed to the well‐recognized higher risk of GI hemorrhage associated with Dabigatran.^42^

### 4.2 Renal adverse drug reactions and fatalities

There is a link between renal clearance pathways and percentage of renal ADRs relative to total prescriptions.^43^ The higher the renal clearance, the higher the percentage of renal reactions. The reasoning being if a DOAC has higher renal clearance then the kidneys are processing a higher amount of drug and are at a greater risk of experiencing a renal ADR especially with declining renal function.^44^ As predicted, Dabigatran having the highest proportion of renal clearance at 80% had the highest rate of renal ADRs out of all DOACs.

The major reported renal ADR extracted from the Yellow Card Scheme for Dabigatran is AKI and Dabigatran had the highest incidence with 1.60 events per 100 000 prescriptions. The cause or nature of the AKI cannot be discerned from the Yellow Card data. However, it has become increasingly apparent that the syndrome of warfarin‐induced nephropathy,that is intraglomerular hemorrhage leading to AKI, also happens with the use of DOACs.^45^ Therefore, DOAC nephropathy is potentially the cause of AKI reported ADRs.

### 4.3 Gastrointestinal adverse drug reactions and fatalities

GI ADRs remain the most commonly reported and well‐documented reaction for the DOACs, in particular, GI hemorrhage.^46^, ^47^, ^48^ DOACs have an increased risk of GI hemorrhage compared to the VKAs with a relative risk of 1.25.^49^ However, no research has defined the underlying cause, other than their mode of action. Suggestions as to why the DOACs cause GI hemorrhage, include: incomplete absorption; and tartaric acid in the Dabigatran formulation causing a direct effect or inhibition of mucosal healing.^42^ Dabigatran is the only DOAC to contain an acid and would be the only DOAC to lead to GI hemorrhage through a direct caustic effect. Dabigatran is associated with the highest percentage of GI ADRs in relation to prescriptions (Table 2). Edoxaban appears to also have a high proportion of GI ADRs relative to prescriptions (Table 2) and this data could be misleading due to the short time the drug has been licensed for and therefore the small number of ADRs and prescriptions potentially artificially inflating the number of reported ADRs in relation to other DOACs.

Our data confirm that Dabigatran is associated with the highest risk of GI hemorrhage. The data also support the fact that Rivaroxaban is the DOAC associated with the second largest risk of GI hemorrhage (Table 2). As Dabigatran is the DOAC with consistently high GI ADRs and incidences of GI hemorrhage throughout preclinical, clinical trial, and postmarketing data, it is unsurprising that it is also associated with the highest percentage of total GI fatalities. It is over 2‐fold that of other DOACs (Table 2) and GI hemorrhage accounts for the highest number of deaths within this organ class. For all DOACs, GI hemorrhage contributes to the highest number of fatal reports within the GI organ class. This suggests that DOACs with the highest risk of GI hemorrhage are also those with the highest risk of GI death.

Dabigatran targets the protease, trypsin (potently), Rivaroxaban (matriptase, modestly) and Apixaban target chymotrypsin (but only at the upper limit of clinical relevance), but importantly, Edoxaban targets neither. Trypsin, chymotrypsin, and matriptase are digestive enzymes found within the GI tract and their secretion leads to mucosal vulnerability that in turn, makes the GIT more prone to gastric bleeding.^50^ Through binding interactions with the DOACs, these enzymes become trapped within the GIT and lead to increased mucosal vulnerability and thus increased rates of GI hemorrhage and fatalities. Apixaban binds with weak affinity; this would explain why the GI hemorrhage risk is not high. Rivaroxaban should be between Apixaban and Dabigatran in terms of risk. Dabigatran's binding affinity to Trypsin is potent (50.3 nmol/L) and along with the tartaric acid present in the formulation, renders the GI mucosa even more vulnerable to hemorrhage,^50^ potentially explaining the high number of reports related to GI ADRs and in particular GI hemorrhage. Dabigatran also has an acidic *p*K_a_, further increasing the vulnerability of the GI mucosa. In addition, Apixaban and Dabigatran are associated with poor bioavailability (Table 1), and as both drugs are absorbed via the GIT, the remainder of the unabsorbed drug remains within the GIT. The drug will have local anticoagulation effects within the GIT, leading to GI ADRs such as GI discomfort, dysphagia, and also GI hemorrhage. As Dabigatran has poor bioavailability at 6.5%, the local effects would be more pronounced than for Apixaban which has a bioavailability of 50%, further explaining the higher percentage of GI ADRs and GI hemorrhage for Dabigatran.

### 4.4 Central nervous system adverse drug reactions and fatalities

Nervous system ADRs are the second most frequent reactions reported across all DOACs within the United Kingdom. Based on the BBB penetrant properties of the four DOACs we predicted that Rivaroxaban would be associated with the highest number of CNS ADRs. However, this was not the case for total CNS ADRs, reported CNS fatalities, all stroke, fatal stroke and hemorrhagic stroke. Indeed, the highest rates were largely seen with Dabigatran which only had one BBB penetrant property compared with Rivaroxaban that had five. It is not clear why this should be, but might be related purely to an enhanced risk of CNS bleeding with Dabigatran. However, our results call into question a simple interpretation of the number BBB penetrant properties to predict CNS ADRs.

### 4.5 Pulmonary embolism

We predicted Dabigatran and Apixaban would be associated with the highest rates of reported pulmonary embolic events. Although this held true for Dabigatran it was not so for Apixaban. Dabigatran and Apixaban, both target tPA, a serine protease that has fibrinolytic activity and can break down blood clots within the body.^40^ It has also been used to treat PE.^39^ The reported tPA data for Apixaban is not specifically documented, however, Dabigatran, has a known affinity of 45 μmol/L. Dabigatran inhibits tPA, inhibiting fibrinolytic activity, resulting in reduced breakdown of blood clots. This could potentially be a reason as to why Dabigatran provides the highest relative amount of PE ADRs compared to the other DOACs.

It should also be noted that all DOACs investigated can be prescribed for PE and stroke, and therefore, these ADR classifications may result from the clinical diagnosis for prescribing the DOAC.

### 4.6 Limitations

Data on the DOACs were obtained using Interactive Drug Analysis Profiles from the MHRA. Drug Analysis Profiles gave a complete listing of all the spontaneous suspected ADRs reported through the Yellow Card Scheme by healthcare professionals, patients, and the pharmaceutical industry in the United Kingdom irrespective of other drugs taken or comorbidities. Covariate data analysis could therefore not be conducted. As such, the data within represents postmarketing phase IV data with real‐world patients. They do not however, present a complete overview of the risks associated with specific medicines. Conclusions on the safety and risks of medicines cannot therefore be made on the information contained in Drug Analysis Profiles alone,^51^ only signal (hypothesis) generation.

In addition, it cannot be concluded that there is a definite causal link between the drug and the adverse patient event that has been reported until further investigations have occurred. It may also be difficult to determine the difference between a reaction that occurred naturally as a result of the disease being treated (eg, PE or stroke) and an ADR. Therefore, only enhanced signals compared to the baseline or where disparate levels of ADRs between the well‐established DOACs were interpreted in this study (Charts S1 and S2).

## 5 CONCLUSIONS

The emergence of DOACs in the late 2000s has altered the treatment for thromboembolic diseases, including AF and stroke. The safety of DOACs has remained an ongoing topic of research due to risks of certain increased bleeds compared to warfarin. We have established a correlation between physicochemical and pharmacological properties, which reflect drug‐target interaction in vivo. This work firstly corroborates the existing literature on GI hemorrhage in DOACs but new findings regarding stroke and AKI can be tentatively linked to the mode of action and selectivity profile. Indeed, three of the five predictions made based on the pharmacological and chemical properties of the DOACs have been supported by interpretation of the UK Yellow Card database and were statistically validated. However, we recommend close monitoring for pulmonary embolism going forward, as current data are limited.

In summary, we propose the use of pharmacological and chemical data as a forward‐looking predictor of drug liabilities in phase IV monitoring, in conjunction with real‐time adverse event reporting, to identify early risks with novel drugs. Furthermore, the predictions made using this data, backed up by the reported ADR data, could potentially be used by clinicians to select the appropriate DOAC for an individual patient.

## DISCLOSURE

The authors report no conflict of interest.

## AUTHOR CONTRIBUTIONS

CJF—analysed and interpreted data, wrote, and revised manuscript; FS—data acquisition, analysis, and interpretation; ZJ—supervision and revised manuscript; AAM—supervision, statistical advice, revised manuscript; AMJ—devised project, supervision, analyzed, and interpreted data, wrote and revised manuscript.

## Supporting information

Table S1‐S2‐Charts S1‐S2

## Data Availability

Data are available in article supplementary material.
