# Supplementary material for: Relevance of physicochemical properties and functional pharmacology data to predict the clinical safety profile of direct oral anticoagulants
Source: Pharmacol Res Perspect. 2020 Jun 4;8(3):e00603. doi: 10.1002/prp2.603 (PMC7272392; doi:10.1002/prp2.603)

ELECTRONIC SUPPORTING INFORMATION

Relevance of physicochemical properties and functional pharmacology data to predict the clinical safety profile of direct oral anticoagulants

C. J. Ferro,[1] F. Solkhon,[2] Z. Jalal,[2] A. M. Al-Hamid,[2] A. M. Jones[2*]

[1] Queen Elizabeth Hospital, University Hospitals Birmingham NHS Foundation Trust, B15 2GW, UK

[2] School of Pharmacy, University of Birmingham, B15 2TT, UK

* A.M.J. +44(0)121-414-7288; a.m.jones.2@bham.ac.uk

Table S1. Reaxy MedChem Module results. Data reported as *p*X values. A threshold of ~10 μM (*p*X 5) for at least one DOAC was used to exclude biologically insignificant off-target interactions.[1]

Biological target	Dabigatran	Rivaroxaban	Apixaban	Edoxaban
Coagulation factor X	6	10.4	10.1	9.2
Coagulation factor X [rabbit]	-	7.6	9.8	9.3
Activated factor [rabbit]	-	9.1	9.7	-
Prothrombinase complex	-	-	8.7	-
Coagulation factor X [dog]	-	-	8.7	-
Fibroleukin	-	-	8	-
Antistasin [human]	-	-	7.3	-
Trypsin [human]	8.1	1	6	-
Thrombin [human]	9.1	8.9	6	5.2
Trypsin-1	7.3	1	5.5	-
Thrombin	8.3	1	5.5	-
Plasma kallikrein	8.5	-	5.4	-
Chymotrypsin B [human]	8.6	-	5.4	-
Tissue-type plasminogen activator	8.4	-	1	-
Plasmin [human]	7.5	1	1	-
Factor IXa [human]	7.4	1	1	-
Solute carrier family 22 member 1	5.3	-	-	-

[1] Miljkovic F and Bajorath J. Data-Driven Exploration of Selectivity and Off-Target Activities of Designated Chemical Probes. *Molecules*. 2018;23.

Table S2. Statistical summary of DOAC ADRs (Results of Chi-squared tests)

**ADR**	**All DOACs**	**Apixaban v Dabigatran**	**Apixaban v Rivaroxaban**	**Apixaban v Edoxaban**	**Dabigatran v Rivaroxaban**	**Dabigatran v Edoxaban**	**Rivaroxaban v Edoxaban**
Total ADRs	<0.001	<0.001	<0.001	<0.001	<0.001	<0.001	0.001
Total Fatalities	<0.001	<0.001	0.153	<0.001	<0.001	0.001	0.002
**Renal System**							
ADR	<0.001	<0.001	<0.001	0.389	<0.001	0.009	0.533
Fatalities	0.756	1.000	0.660	1.000	1.000	N/A	1.000
AKI	<0.001	<0.001	0.302	0.626	<0.001	0.018	0.641
**Gastrointestinal System**							
ADR	<0.001	<0.001	<0.001	<0.001	<0.001	<0.001	0.033
Fatalities	<0.001	<0.001	0.147	1.000	<0.001	0.084	1.000
GI haemorrhage	<0.001	<0.001	<0.001	0.008	<0.001	<0.001	0.912

Fatal GI haemorrhage	<0.001	<0.001	0.220	1.000	<0.001	0.084	1.000
**Central Nervous System**							
ADR	<0.001	<0.001	<0.001	<0.001	<0.001	0.601	0.016
Fatalities	0.005	0.012	0.001	1.000	0.648	0.474	0.639
Stroke	<0.001	<0.001	0.199	0.003	<0.001	0.727	0.001
Fatal stroke	0.742	0.571	0.859	1.000	0.383	1.000	1.000
Haemorrhagic stroke	0.403	0.821	0.271	1.000	0.426	1.000	0.631
**Respiratory System**							
ADRS	<0.001	<0.001	<0.001	0.779	<0.001	<0.001	0.076
Fatalities	<0.001	<0.001	0.053	1.000	<0.001	0.625	1.000
Pulmonary embolism	<0.001	<0.001	<0.001	1.000	<0.001	0.005	0.591

Chart S1. Number of reported ADRs within the UK per Organ Class per DOAC.

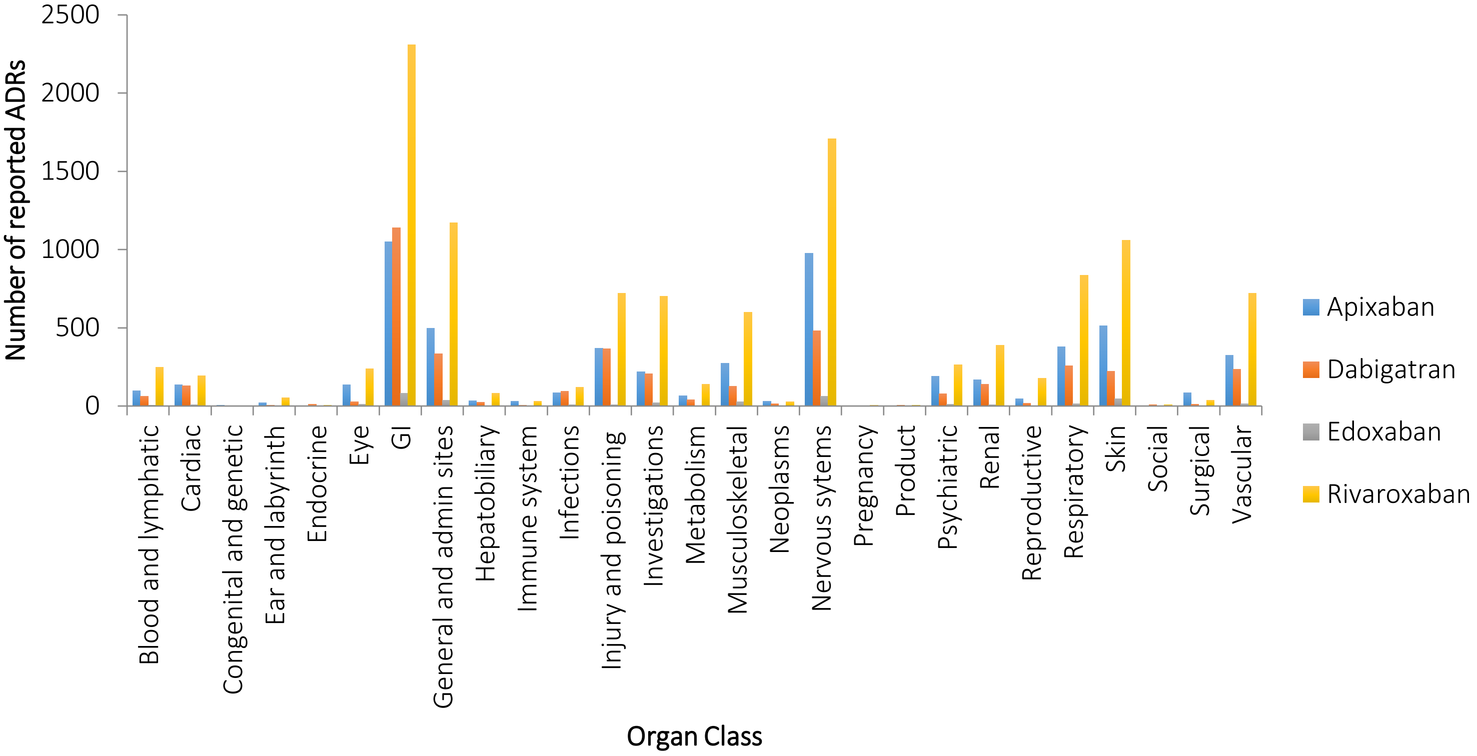

Chart S2. Total number of reported fatalities within the UK per organ class for each of the four DOACs.

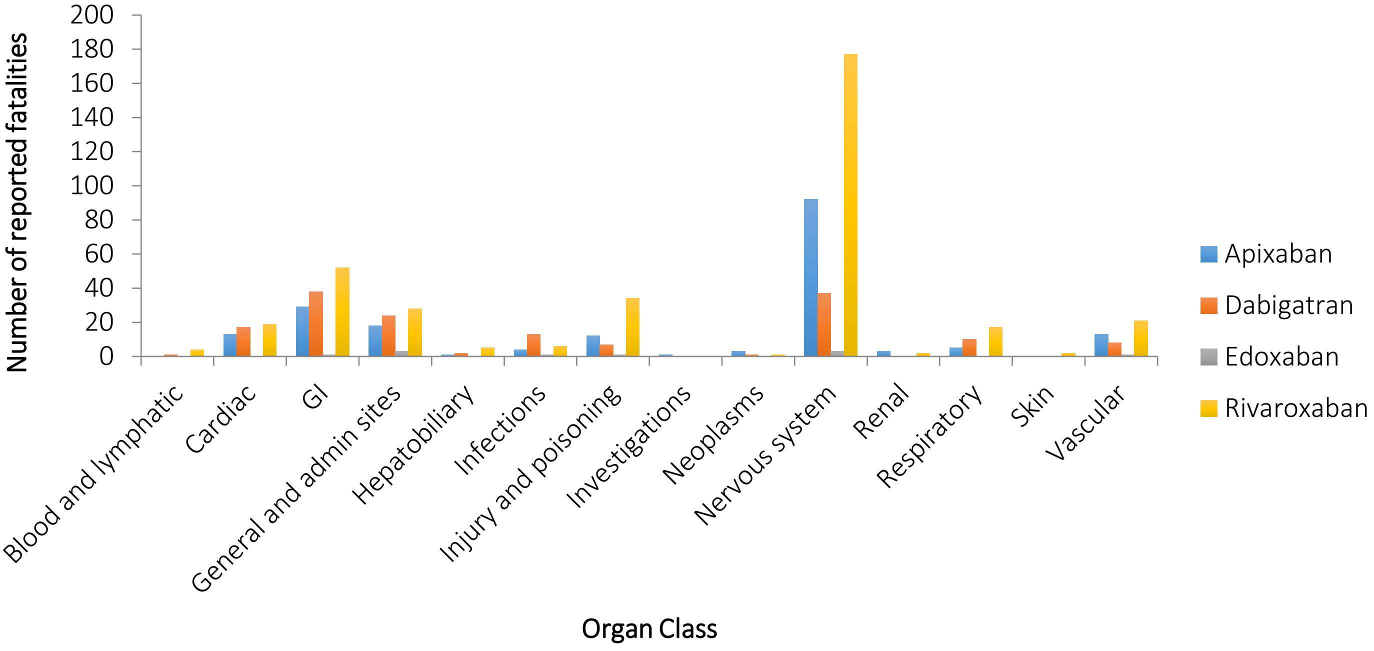

Supplement: Supplementary file 1 — Table S1‐S2‐Charts S1‐S2 [file PRP2-8-e00603-s001.pdf]
